# Co-targeting the kappa opioid receptor and dopamine transporter reduces motivation to self-administer cocaine and partially reverses dopamine system dysregulation

**DOI:** 10.1038/s41598-024-53463-9

**Published:** 2024-03-18

**Authors:** Paige M. Estave, Steven E. Albertson, Anushree N. Karkhanis, Sara R. Jones

**Affiliations:** 1 Department of Physiology and Pharmacology, Wake University Forest School of Medicine, Medical Center Blvd., Winston-Salem, NC 27157 USA; 2 Department of Psychology, Binghamton University – State University of New York, Binghamton, NY 13902 USA

**Keywords:** Cocaine, Dopamine, Kappa, Dopamine transporter, Self-administration, Combination drug therapy, Addiction, Neurophysiology

## Abstract

Cocaine disrupts dopamine (DA) and kappa opioid receptor (KOR) system activity, with long-term exposure reducing inhibiton of DA uptake by cocaine and increasing KOR system function. Single treatment therapies have not been successful for cocaine use disorder; therefore, this study focuses on a combination therapy targeting the dopamine transporter (DAT) and KOR. Sprague Dawley rats self-administered 5 days of cocaine (1.5 mg/kg/inf, max 40 inf/day, FR1), followed by 14 days on a progressive ratio (PR) schedule (0.19 mg/kg/infusion). Behavioral effects of individual and combined administration of phenmetrazine and nBNI were then examined using PR. Additionally, ex vivo fast scan cyclic voltammetry was then used to assess alterations in DA and KOR system activity in the nucleus accumbens before and after treatments. Chronic administration of phenmetrazine as well as the combination of phenmetrazine and nBNI—but not nBNI alone—significantly reduced PR breakpoints. In addition, the combination of phenmetrazine and nBNI partially reversed cocaine-induced neurodysregulations of the KOR and DA systems, indicating therapeutic benefits of targeting the DA and KOR systems in tandem. These data highlight the potential benefits of the DAT and KOR as dual-cellular targets to reduce motivation to administer cocaine and reverse cocaine-induced alterations of the DA system.

## Introduction

Addiction is an ongoing public health crisis, with opioids and cocaine leading the list of drugs responsible for overdose deaths in the United States. Notably, from 2015 to 2021, there was a 2.5-fold increase in overdose deaths involving cocaine^1^. However, the FDA has yet to approve any medication to treat cocaine use disorder. Therefore, a greater understanding of cocaine’s mechanisms and the neurobiological alterations induced by chronic cocaine exposure is needed to inform medication development.

Cocaine functions as a reinforcer, acting primarily through inhibition of the dopamine transporter (DAT)^2–7^, which leads to elevated extracellular dopamine (DA) levels, particularly in limbic regions of the brain^8–13^. After chronic cocaine exposure, extensive compensatory neuroadaptations lead to deficits in DA signaling, especially during drug withdrawal^14–17^. PET studies in individuals with cocaine use disorder show an association between low D1 receptor availability in the ventral striatum and increased cocaine choices over monetary vouchers^18^. Additionally, several PET studies have revealed persistent reductions in striatal D2/D3 receptor binding in individuals with cocaine use disorder^19–21^ (but see Ref.^22^). Postmortem striatal tissue from chronic cocaine users showed increased DAT binding and increased DAT function^23,24^, which would tend to reduce extracellular DA levels. Overall, these changes point to a hypodopaminergic state in humans after chronic cocaine use^25–27^, which is also seen in preclinical models of cocaine addiction^28–32^.

Prevailing theories of addiction suggest that positive reinforcement drives initial drug use, whereas negative reinforcement, aimed at alleviating withdrawal-associated negative affective states (anxiety, dysphoria, anhedonia), promotes later compulsive drug use^33–35^. Changes in DA over time mirror this switch in reinforcement valence, with early transient cocaine-induced DA elevations preceding long-term reductions in signaling and drastically reduced cocaine effects (for review see Refs.^36,37^). During early drug use, humans report that cocaine initiates a feeling of euphoria, or “high”^38–40^; however, after long-term use, subjects no longer take cocaine to get “high” but to reduce drug craving and feelings of dysphoria and anhedonia—allowing them to feel somewhat “normal” while under the influence of cocaine^41,42^ (for review see Ref.^43^).

In preclinical animal models, chronic cocaine exposure induces DA system adaptations that indicate reduced DA function, including (1) low tonic levels, (2) reduced effects of cocaine on extracellular DA levels, (3) decreased DA response to environmental stimuli in vivo, (4) reduced DA release after electrical stimulation in ex vivo preparations, and (5) tolerance to cocaine’s inhibitory effects on the DAT^32,44–47^. To address the state of low DA after chronic use, many putative medications for cocaine use disorder have targeted DATs. DA “releasers”, for example, competitively bind to DATs, inhibit uptake, reverse the direction of transport, and promote non-vesicular release of DA into the extracellular space^48–52^. Releasers have been shown to reduce voluntary cocaine intake in both clinical and preclinical studies, including in our own laboratory (amphetamine, Refs.^53–58^; phenmetrazine/phendimetrazine, Refs.^59,60^), (amphetamine, Refs.^47,61^; phenmetrazine, Ref.^62^).

In addition to DA deficits, recruitment of brain stress systems, including the corticotrophin-releasing factor and kappa opioid receptor (KOR) systems, evoke negative affective states that intensify the negative reinforcing aspects of ongoing drug use^63–65^. In this study, we focus on KORs, which are widely distributed throughout the brain, with a high density in the nucleus accumbens (NAc) where they are located on both neuronal cell bodies and afferent axon terminals, including DA terminals^66,67^. When DA terminal KORs are activated, DA release is inhibited, contributing to a low-functioning DA system (hypodopaminergia)^62,66,68–71^ (but see Refs.^72,73^). This inhibition of DA release promotes anxiety- and depressive-like behaviors^64,65,74^, which may heighten the risk of relapse in cocaine-dependent individuals. Conversely, KOR antagonists reduce anxiety-like behaviors and prevent stress-induced reinstatement of cocaine-seeking^75–80^. Repeated cocaine exposure in animal models^81–83^ and in humans^84–86^ leads to an upregulation of KORs in the NAc, which is thought to reduce DA transmission. A recent study using PET imaging revealed a positive correlation between cocaine choice and KOR binding (availability) in many brain regions, including the ventral striatum, following an acute stressor^87^. For these reasons, KOR antagonists may be beneficial in reducing KOR activity and restoring extracellular levels of DA after chronic cocaine exposure.

Enduring changes in DA and KOR systems after chronic cocaine exposure likely drive, at least in part, escalation of drug intake as well as increased seeking and relapse-like behaviors, presumably in an attempt to restore DA homeostasis. This study, for the first time, examines the combined effects of the DA releaser phenmetrazine and the KOR antagonist norbinaltorphimine (nBNI) on the motivation to take cocaine and dopamine system dysregulations. In this preclinical study, we find putative benefit of combination therapy with phenmetrazine and nBNI, which is echoed by a wealth of clinical literature indicating several advantages of combination therapy, including the potential to treat multiple disease symptoms^88–90^. This is particularly important for a complex disease such as cocaine use disorder, which is notoriously difficult to treat.

## Materials/Methods

### Animals

Male Sprague–Dawley rats weighing 325–400 g at time of delivery to our laboratory (Envigo, Indianapolis, IN) were used as subjects and were maintained according to the NIH guidelines in Association for Assessment and Accreditation of Laboratory Animal Care accredited facilities. Housing facilities were kept on a 12:12 h reversed cycle of light and dark, with lights coming on at 1500 h. Food and water were given ad libitum*.* Animals were excluded from the study if they lost catheter patency or showed signs of infection before completing all phases of the study (n = 8 lost catheter patency; n = 1 infection). One additional animal was removed due to erroneously being given the wrong cocaine dose. The protocol was approved by Wake Forest University School of Medicine’s Institutional Animal Care and Use Committee. All methods were performed in accordance with the relevant guidelines and regulations. The study is reported in accordance with ARRIVE guidelines.

### Drugs

For behavior, Cocaine HCl (National Institute on Drug Abuse Drug Supply Program, Bethesda, MD) and phenmetrazine hemifumarate (RTI International, Research Triangle Park, NC) were dissolved in sterile saline; norbinaltorphimine (nBNI) was dissolved in sterile injectable water. For fast-scan cyclic voltammetry, the KOR agonist U50,488 (National Institute on Drug Abuse Drug Supply Program, Bethesda, MD) and Cocaine HCl were dissolved in deionized water.

### Catheter implantation

After being anesthetized with ketamine (100 mg/kg, i.p.) and xylazine (8 mg/kg, i.p.), rats were implanted with a chronic indwelling jugular catheter (see Ref.^91^ for further details). Post-surgery, animals were given 5 mg/kg ketoprofen subcutaneously for postoperative analgesia. They were then individually housed in 30 × 30 × 30 cm custom-made operant conditioning chambers where self-administration sessions took place in the active/dark cycle (0900h–1500h). Control animals for fast-scan cyclic voltammetry experiments were implanted with a closed-off port on the animal’s dorsum to control for housing and surgery conditions.

### Cocaine self-administration training

On the third day after surgery, rats were placed on a fixed-ratio one (FR1) schedule of reinforcement without any prior operant training. When the lever was pressed, the animal received a single intravenous infusion of cocaine (1.5 mg/kg/infusion) over approximately 4 s. During the infusion, the lever retracted and a stimulus light above the lever illuminated for the duration of the infusion. Sessions were terminated after 40 infusions or 6 h elapsed, whichever occurred first. Animals were required to complete 5 consecutive days of 40 infusions before advancing to the next part of the paradigm, progressive ratio (PR) schedule of reinforcement, which took on average 2 weeks.

### Progressive ratio schedule of reinforcement

Once training was complete, the schedule was switched to PR (0.19 mg/kg/infusion). Response requirements increased in the following ratio sequence: 1, 2, 4, 6, 9, 12, 15, 20, 25, 32, 40, 50, 62, 77, 95, 118, etc.^62^. The breakpoint was defined as the “number of reinforcers earned before one hour elapsing without completion of the next response requirement”. Breakpoint was used as the outcome measure for all statistical analyses (see Ref.^92^ for details).

### Cocaine self-administration paradigm

The behavioral portion of this study aimed to examine the individual and combined effects of phenmetrazine and nBNI on PR responding (Figs. 2, 3). Once responding on PR was stable (number of infusions earned did not differ by more than 20% over three days, with no upward/downward trend; approximately 4–7 days), animals were treated with phenmetrazine (25 mg/kg/day, s.c. osmotic minipump, Model 2001 Alzet, Cupertino, CA; n = 26), nBNI (10 mg/kg, ip; n = 10) or saline (n = 6) to determine therapeutic effects of each monotherapy. Another group of animals received phenmetrazine + nBNI (n = 9). Osmotic minipumps were replaced every 7 days as needed, and injections of nBNI were administered every 7 days. nBNI is known to be a long-lasting KOR antagonist (see Refs.^93,94^ for details), with a single injection resulting in behavioral effects for up to 3 weeks^93,95–97^. Once responding was stable, animals received a second phase of treatment to determine effects over a longer treatment period. Breakpoints were recorded when behavior was stable (approximately 4–7 days).

### Food self-administration training

A separate group of animals were used for food self-administration. To control for housing conditions and surgery, animals underwent surgery to implant a closed-off port on the animal’s dorsum to allow animals to be tethered in its home cage (similar to cocaine self-administering animals). Two days after surgery, rats were placed on an FR1 schedule of reinforcement without any prior operant training or food deprivation. Each press on the active lever resulted in the delivery of a single 45-mg chocolate-flavored rat chow pellet (Bio-Serv, Flemington, NJ; product # F0299, dustless precision pellets, purified diet). When a reinforcer (pellet) was received, the lever was retracted and a stimulus light above the lever was illuminated for a 20-s timeout period. Responses on the inactive lever were recorded. Sessions were terminated after 40 reinforcers were reached or 6 h elapsed, whichever occurred first. Once 5 days of 40 food pellets were earned, conditions were switched to a PR schedule of reinforcement. During PR, the response requirement systematically increased as described above. Once responding on PR was stable (described above), animals were treated with phenmetrazine (25 mg/kg/day, s.c. osmotic minipump, ALZET model 2002; n = 9), nBNI (10 mg/kg, i.p.; n = 8), or the combination of the two treatments for 14 days (n = 8), as described above.

### Ex vivo fast scan cyclic voltammetry (FSCV)

Ex vivo FSCV was then used to assess alterations in DA dynamics in the NAc core after cocaine self-administration training and PR. Approximately 18 h after the end of the final self-administration session (0900 h-1000 h), animals were rapidly decapitated and brains extracted. 400 µM thick coronal brain slices containing the NAc core were prepared using a vibrating tissue slicer (see Ref.^62^ for details). Once slices were transferred to recording chambers, a carbon fiber microelectrode (150–200 μM length, 7 μM diameter) and a bipolar stimulating electrode were placed into the NAc core. DA release was evoked every 5 min by applying a single electrical pulse from the bipolar stimulating electrode (350 μA, 4 ms, monophasic) to the tissue. The DA concentration was recorded by applying a triangular waveform (− 0.4 to + 1.2 to − 0.4 V vs. Ag/AgCl, at a rate of 400 V/s) to the carbon fiber microelectrode. Once the evoked release of DA was stable, drug concentration–response curves were obtained by adding drugs cumulatively to the superfusion buffer once the effect of each drug reached stability (~ 45 min). Drug concentrations were as follows: Cocaine 0.30, 1.0, 3.0, 10.0, 30.0 μM and U50,488 0.01, 0.03, 0.10, 0.30, 1.0, 3.0 μM.

### Data analysis

To evaluate DA release and uptake kinetics, the program Demon Voltammetry and Analysis was used for analysis^98^. To calibrate recording electrodes, a current response to a known concentration of DA was used (our laboratory uses 3 uM). This was then converted from the output current of the recording electrode (nA) to a DA concentration (uM). Michaelis–Menten based modeling in Demon Voltammetry and Analysis is then used to determine the amount of stimulated DA release, the maximal rate of DA uptake (*V*_max_), and the ability of DA to bind to the DAT in the presence of the competitive inhibitor cocaine (apparent *K*_m_). See^98^ for further details.

### Statistical analysis

GraphPad Prism 8 (Graph Pad Software, La Jolla, CA) was used to conduct all analyses. Data is reported as mean ± standard error, and the significance level was set at p < 0.05.

#### Behavior

A repeated measures one-way analysis of variance (ANOVA) was conducted to compare the effects of treatment phase. A one-way ANOVA was then used to compare phase 2’s treatment response to control animals with saline minipumps. ANOVAs were followed by planned comparisons using Tukey’s multiple comparisons test. Paired t-tests were used to compare breakpoints for food responding.

#### Voltammetry

A Student’s t-test was conducted to compare baseline DA release and uptake rate between groups. Concentration–response curves were subjected to a repeated measures two-way ANOVA, with concentration as the within-subject factor and experimental group as the between-subjects factor. DA release (U50,488 experiments) or apparent Km (cocaine experiments) were the dependent variable. The ANOVAs were followed by targeted pairwise comparisons between groups using a Sidak post hoc test. Due to unequal variance between groups, a Welch’s t-test was utilized to compare IC50s.

## Results

### Prior cocaine exposure decreases DA transporter inhibition by cocaine and increases kappa opioid receptor activity in the NAc

**Figure 1:**
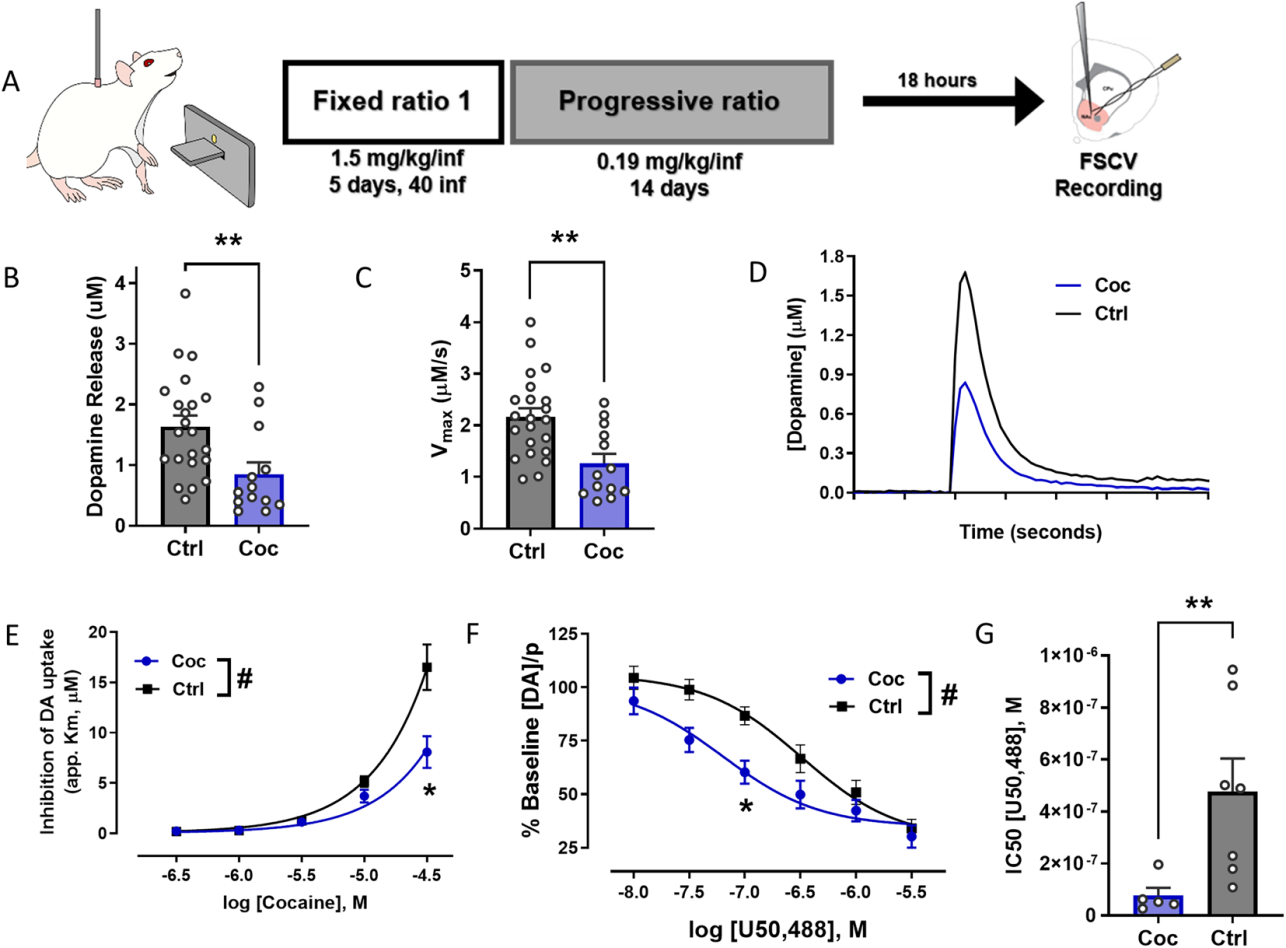
Decreased DAT response to cocaine and heightened KOR function in rats after cocaine self-administration. (**A**) Schematic diagram of experimental timeline for animals self-administering cocaine. (**B**) Average evoked DA release in the cocaine group (blue, Coc, n = 13) compared to naïve controls (grey, Ctrl, n = 22). (**C**) Average maximal uptake rate (V_max_). (**D**) Averaged raw data traces illustrating differences in evoked release and uptake. (**E**) Group data showing that the cocaine group exhibited a reduction in response of the DAT to cocaine (Ctrl n = 8, Coc n = 6), and (**F**) an increase in response to U50, 488, a KOR agonist (Ctrl n = 7, Coc n = 5). (**G**) Increased potency of U50, 488 in the cocaine group, defined by the half-maximal Inhibitory Concentration (IC50). *p < 0.05, **p < 0.01, ***p < 0.001, ****p < 0.0001. Main effect: #p < 0.05, ##p < 0.01. Data shown as mean ± SEM. *FSCV* fast-scan cyclic voltammetry, *inf* infusion, *coc* cocaine, *ctrl* control, *DAT* dopamine transporter, *DA* dopamine, *KOR* kappa opioid receptor.

Our laboratory has previously shown that 5 days of 40 cocaine infusions (1.5 mg/kg/inf) results in a decrease in cocaine’s ability to inhibit DA uptake at the DAT^13,45,46^. Here, the effects of an additional 14 days of PR (0.1875 mg/kg/infusion) on DA terminal function, cocaine potency, and KOR responsivity were tested using ex vivo FSCV recordings in the NAc core (Fig. 1A).

Compared to naïve controls, cocaine-exposed animals exhibited significantly reduced baseline release (t_(33)=_2.835, p = 0.0078; Fig. 1B,D) and slower rates of maximal uptake (V_max_; t_(33)_ = 3.398, p = 0.0018; Fig. 1C). A repeated measures two-way ANOVA revealed a main effect of cocaine concentration (F_(1.134, 13.61)_ = 57.07, p < 0.0001), cocaine exposure (F_(1, 12)_ = 8.905, p = 0.0114, Fig. 1E), and an interaction between cocaine concentration and cocaine exposure (F_(4, 48)_ = 7.324, p = 0.0001). Post hoc testing revealed a significant effect at the 30uM cocaine concentration (*p* = 0.0498). In addition, comparison of KOR function in control and cocaine-exposed rats using a mixed-effects model with repeated measures showed a main effect of U50,488 concentration (F_(2.213, 20.80)_ = 107.5, p < 0.0001) and cocaine history (F_(1, 10)_ = 6.012, p = 0.0341; Fig. 1F), as well as an interaction between U50,488 concentration and cocaine exposure (F_(5, 47)_ = 2.914, p = 0.0226), indicating that cocaine exposure increases KOR system function in the NAc core. Post hoc testing revealed a significant effect at 0.1 µM U50,488 concentration (p = 0.0259). Welch’s t-test revealed a significant difference in the IC50 of U50,488 between the cocaine and control groups (t_(6.684)=_3.077, p = 0.0084; Fig. 1G).

### Dual-targeting the DAT and KOR decreases cocaine breakpoints on PR

**Figure 2:**
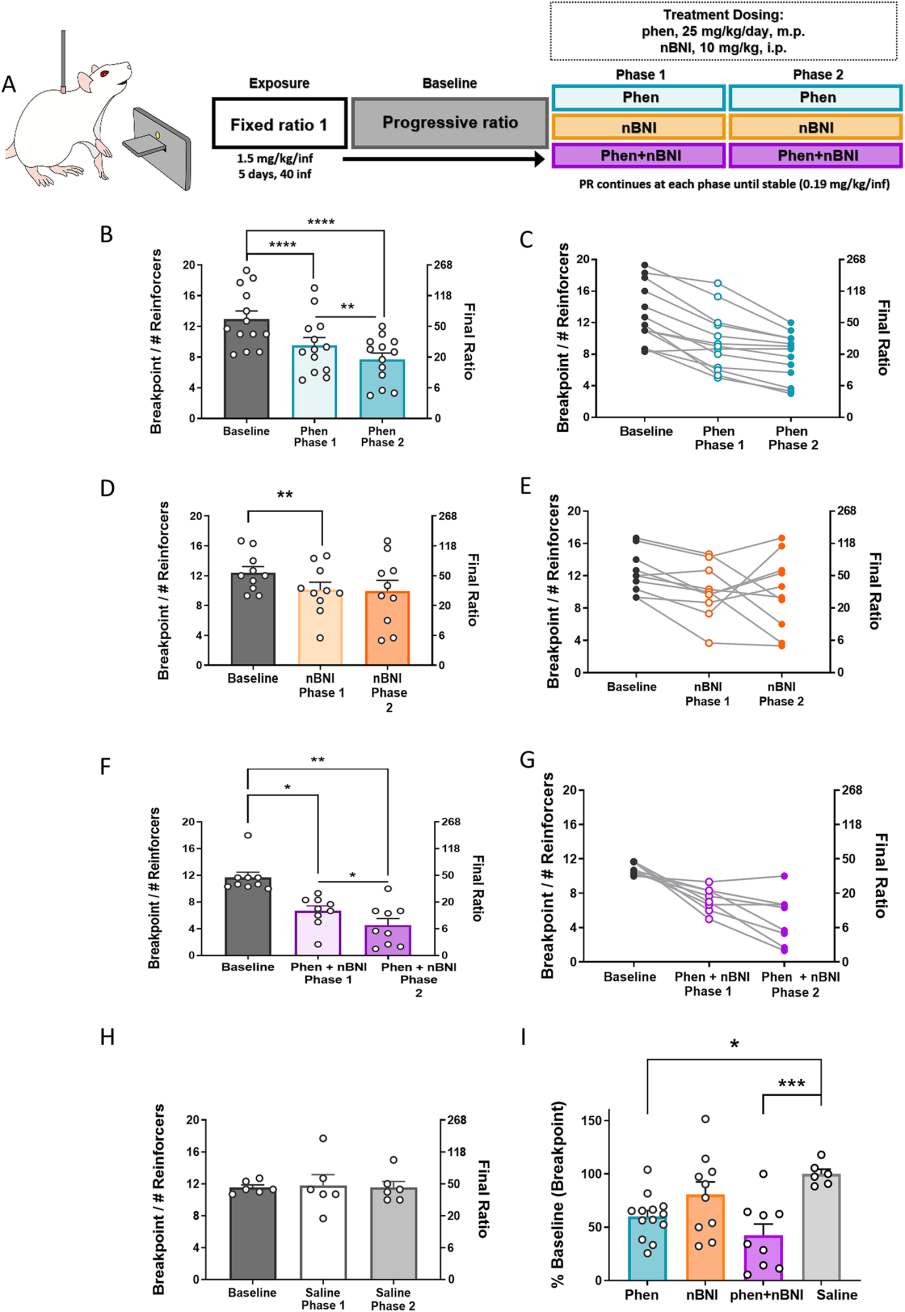
Combination of phenmetrazine and nBNI decreased number of cocaine infusions earned on a PR schedule of reinforcement. (**A**) Schematic diagram of experimental timeline. (**B**) Average breakpoint at baseline (grey), after Phase 1 (light teal), and after a second phase of phenmetrazine treatment (dark teal). (**C**) Before-after graph showing individual changes. (**D**) Average breakpoint at baseline (grey), after Phase 1 (light orange), and after a second phase of nBNI treatment (dark orange). (**E**) Before-after graph showing individual changes. (**F**) Average breakpoint at baseline (grey), after Phase 1 (light purple), and after a second phase of phenmetrazine + nBNI treatment (dark purple). (**G**) Before-after graph showing individual changes. (**H**) Average breakpoint at baseline (dark grey), and after saline minipump treatment. (**I**) Average change from each treatment depicted as a percent of baseline. Phenmetrazine n = 13, nBNI n = 10, Phen + nBNI n = 9, saline n = 6. *p < 0.05, **p < 0.01, ***p < 0.001, ****p < 0.0001. Data shown as mean ± SEM. *PR* Progressive ratio, *Phen* phenmetrazine, *nBNI* norbinaltorphimine, *inf* infusion, *m.p.* osmotic minipump, *i.p.* intraperitoneal.

Because cocaine self-administration markedly changed the function of both DATs and KORs (Fig. 1), we tested the effects of the DA releaser phenmetrazine and the KOR antagonist nBNI on cocaine breakpoints (Fig. 2). If DA or KOR system dysregulations are involved in the reinforcing effects of cocaine, treatment with phenmetrazine or nBNI should alter cocaine breakpoints on a PR schedule. To test this hypothesis, animals self-administered cocaine for 5 days (FR1, 40 infusions max, 1.5 mg/kg/inf) to induce changes to the DA and KOR systems, as described above. After this exposure, PR commenced (0.19 mg/kg/inf) until breakpoints were stable. A minipump containing phenmetrazine (25 mg/kg/inf) was implanted subcutaneously, an i.p. injection of nBNI (10 mg/kg) was given, or a combination of the two treatments was administered. See Fig. 2A for experimental timeline. Both phenmetrazine and nBNI decreased breakpoints in the first phase of treatment, suggesting a role of DATs and KORs in the reinforcing effects of cocaine. In the phenmetrazine group (n = 13), breakpoints decreased to 73.2% ± 4.44 of baseline during phase 1, with a further decrease to 59.9% ± 5.69 of baseline during phase 2. There was a significant effect of treatment phase (F_(1.358,16.3)_ = 43.33, p < 0.0001; Fig. 2B), with a significant decrease in breakpoint from baseline to phase 1 (p < 0.0001) and from phase 1 to phase 2 (p = 0.0016). In the nBNI group (n = 10), breakpoints decreased to 80.4% ± 5.70 of baseline in phase 1; in phase 2, breakpoints remained stable at 80.6% ± 12.02 of baseline. There was no significant effect of treatment phase (F_(1.211, 10.9)_ = 2.503, p = 0.1396; Fig. 2D), with a significant decrease from baseline to phase 1 of nBNI treatment (p = 0.0076). In the phen + nBNI group (n = 9), breakpoints decreased to 61.37% ± 8.33 of baseline in phase 1; in phase 2, breakpoints further decreased to 42.5% ± 10.49 of baseline. There was a significant effect of treatment phase (F_(1.276, 10.21)_ = 14.26, p = 0.0024; Fig. 2F), with a significant decrease in breakpoint from baseline to phase 1 (p = 0.0292) and from phase 1 to phase 2 in the combination group (p = 0.0353). There was no effect of treatment in animals with saline minipumps (F_(1.171, 5.857)_ = 0.06, p = 0.8505; Fig. 2H). When comparing each group’s breakpoints during phase 2 to their respective baselines, there was a main effect of treatment (F_(3, 34)_ = 6, p = 0.0018; Fig. 2I), with a significant decrease in the phenmetrazine (p = 0.0306) and phen + nBNI (p = 0.0023) groups.

### Monotherapy with phenmetrazine or nBNI, or combination of the two, does not decrease food responding on a progressive ratio schedule of reinforcement

**Figure 3:**
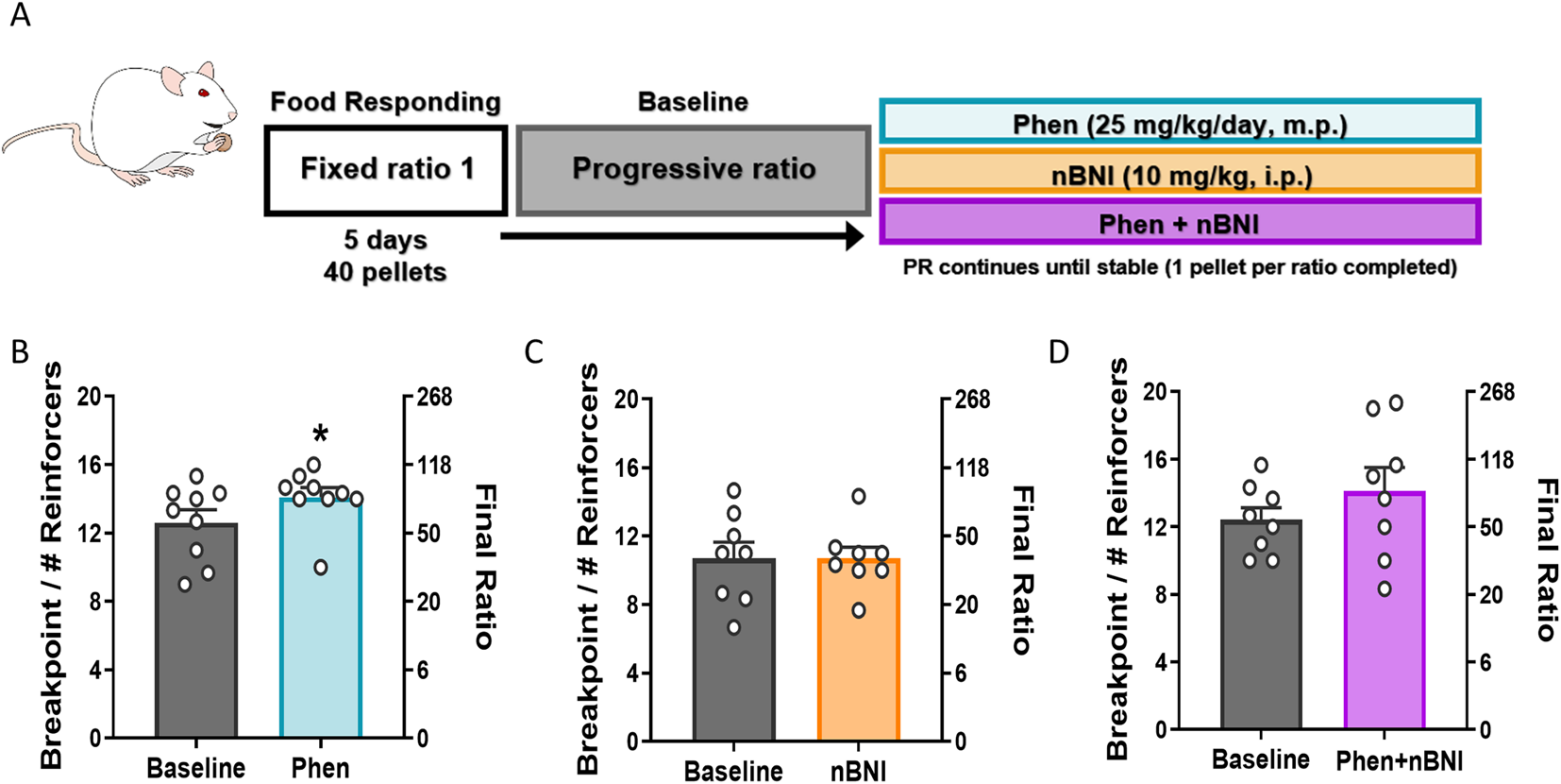
Neither phenmetrazine alone, nBNI alone, nor a combination of the two decreased the number of palatable food pellets earned on a PR schedule of reinforcement. (**A**) Schematic diagram of experimental timeline. (**B**) Average breakpoint at baseline (grey) and after phenmetrazine treatment (teal). (**C**) Average breakpoint at baseline (grey) and after nBNI treatment (orange). (**D**) Average breakpoint at baseline (grey) and after phenmetrazine + nBNI treatment (purple). Phenmetrazine only n = 9, nBNI only n = 8, Phenmetrazine + nBNI n = 8. Data shown as mean ± SEM. *PR* progressive ratio, *Phen* phenmetrazine, *nBNI* norbinaltorphimine.

Given the impact of treatments on cocaine breakpoints, we wanted to assess the specificity of this effect for cocaine vs a non-drug reinforcer. Therefore, we trained three groups of animals to self-administer palatable chocolate pellets (Fig. 3A). In the three groups of rats self-administering food, there was no significant difference in the number of pellets received at baseline (data not shown; F_(2, 13)_ = 0.4794, p = 0.6297). Treatment with phenmetrazine (n = 9; 25 mg/kg/day, minipump) increased food maintained responding (t_(8)_ = 2.365; p = 0.0456; Fig. 3B). However, nBNI (n = 8; 10 mg/kg, i.p; t_(7)_ = 0.0; p > 0.9999; Fig. 3C), or a combination of the two (n = 8; t_(7)_ = 2.211; p = 0.0627; Fig. 3D), did not alter food breakpoints on PR. This suggests that the decrease in cocaine breakpoint seen in Fig. 2 is not due to general suppression of behavior and that the effects of these drugs are not generalizable to all reinforcers. In addition, there was no significant difference between the number of food pellets and number of cocaine injections earned at baseline, suggesting that the food pellets used here and cocaine (0.19 mg/kg/inf) have comparable reinforcing strength.

### Treatment with phenmetrazine and nBNI partially reverse cocaine-induced dysregulations of the DA system

**Figure 4:**
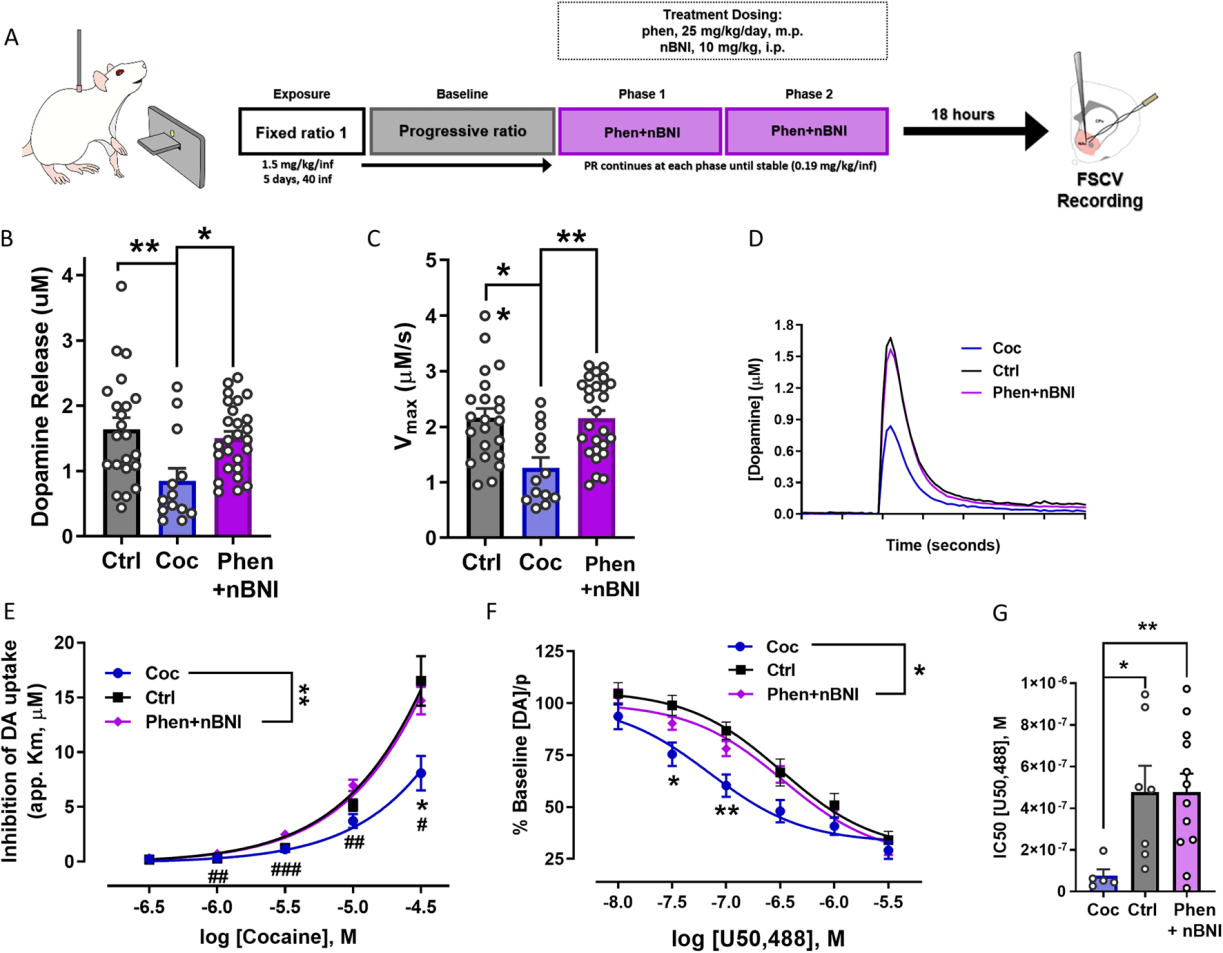
Treatment with phenmetrazine and nBNI reverse cocaine-induced dysregulations of the DA system. (**A**) Schematic diagram of experimental timeline for animals self-administering cocaine. (**B**) Average evoked DA release in the cocaine group (blue, Coc, n = 13), naïve controls (grey, Ctrl, n = 22), and nBNI + phenmetrazine group (purple, Phen + nBNI, n = 26) (**C**) Average maximal uptake rate (V_max_). (**D**) Averaged raw data traces illustrating differences in evoked release and uptake. (**E**) Group data showing that the cocaine group exhibited a reduction in response of the DAT to cocaine, which was blocked by phen + nBNI treatment (Ctrl n = 8, Coc n = 6, phen + nBNI n = 14), and (**F**) an increase in response to U50, 488, a KOR agonist that was partially reversed by phen + nBNI treatment (Ctrl n = 7, Coc n = 5, phen + nBNI n = 12). (**G**) Increased potency of U50, 488 in the cocaine group, defined by the half-maximal Inhibitory Concentration (IC50), that was normalized by nBNI + phen treatment. *p < 0.05, **p < 0.01, ***p < 0.001, ****p < 0.0001. Main effect: ^#^p < 0.05, ^##^p < 0.01. Data shown as mean ± SEM. *Ctrl* control, *coc* cocaine, *FSCV* fast-scan cyclic voltammetry, *DAT* dopamine transporter, *DA* dopamine, *KOR* kappa opioid receptor, *phen* phenmetrazine, *nBNI* norbinaltorphimine, *m.p.* osmotic minipump, *i.p.* intraperitoneal.

As shown in Fig. 1, 5 days of 40 cocaine infusions (1.5 mg/kg/inf), followed by 14 days of PR (0.19 mg/kg/inf) results in a decrease in cocaine’s ability to inhibit DA uptake at the DAT (decreased apparent Km) and an increase in KOR activity in the NAc core. To determine if the combination of phenmetrazine and nBNI can reverse these dysregulations after cocaine self-adminsitration, animals treated with the combination were sacrificed for FSCV (Fig. 4A).

There was a significant main effect of treatment on baseline DA release (F_(2, 58)_ = 5.692, p = 0.0055; Fig. 4B). Compared to naïve controls, cocaine exposed animals exhibited significantly reduced baseline release (p = 0.0053; Fig. 4B), which was significantly increased with nBNI + phen treatment (p = 0.0194; Fig. 4B). Similarly, there was a significant main effect of treatment on baseline maximal uptake rate (F_(2, 58)_ = 7.831, p = 0.0010; Fig. 4C). Compared to naïve controls, cocaine exposed animals exhibited significantly reduced baseline uptake rate (p = 0.0022; Fig. 4C), which was significantly increased with nBNI + phen treatment (p = 0.0017; Fig. 4C). These results suggest that the combination of nBNI and phenmetrazine can reverse and normalize the cocaine-induced changes in baseline dopamine kinetics. Figure 4D demonstrates the averaged raw data traces illustrating differences in evoked release and uptake.

A repeated measures two-way ANOVA revealed a main effect of cocaine exposure on DA uptake inhibition by cocaine (F_(2, 25)_ = 6.876, p = 0.0042, Fig. 4E). Post hoc testing revealed a significant effect between cocaine animals and controls (p = 0.0178; Fig. 4E) as well as cocaine animals and phen + nBNI animals at the 30uM cocaine concentration (p = 0.0117; Fig. 4E). In addition, comparison of KOR function in control, cocaine exposed, and nBNI + phen rats using a repeated measures two-way ANOVA showed a main effect of cocaine exposure on inhibition of DA release induced by U50,488 (F_(2,21)_ = 3.626, p = 0.0444; Fig. 4F). Post hoc testing revealed a significant effect at 0.1 µM U50,488 concentration between cocaine and control animals (p = 0.0076; Fig. 4E) and cocaine animals and phen + nBNI animals (p = 0.0429; Fig. 4F). Welch’s ANOVA test revealed a main effect of treatment on the IC50 of U50,488 between the cocaine and control groups (F_(2.000,11.61)=_12.20, p = 0.0014; Fig. 4G), with a significant decrease in IC50 between cocaine and control groups (p = 0.0486; Fig. 4G) that was significantly increased back to control levels after nBNI + phen treatment (p = 0.0027; Fig. 4G). Here we show that phenmetrazine and nBNI can reverse cocaine-induced DA system changes including (1) baseline release and uptake rates; (2) decreased effects of cocaine at the DAT; and (3) heightened KOR activity.

## Discussion

The present study sought to gauge KOR and DAT activity in the nucleus accumbens after cocaine self-administration and evaluate the individual and combined effects of the DA releaser phenmetrazine and the KOR antagonist nBNI on cocaine breakpoints and DA system dysregulations. Cocaine self-administration resulted in decreased inhibition of DA uptake and increased KOR function after 5 days of 40 infusions/day of high dose cocaine (1.5 mg/kg/inf) followed by 14 days of PR at a low dose (0.1875 mg/kg/inf), which returned to control levels after animals were treated with the combination of phenmetrazine and nBNI. Treatment with phenmetrazine alone (25 mg/kg/day, minipump), a single injection of nBNI (10 mg/kg, i.p.), or a combination of the two significantly reduced cocaine breakpoints. A second phase of treatment continued to decrease breakpoints in the phenmetrazine and phen + nBNI groups, but not the nBNI group. Importantly, phenmetrazine, nBNI, or the combination of the two did not suppress food-maintained responding. Therefore, the reduction in breakpoints observed is specific to the reinforcing effects of cocaine. Furthermore, the FSCV data provided supports a potential underlying mechanism driving these changes in cocaine reinforcement.

### Continuous phenmetrazine treatment is sufficient to reduce cocaine breakpoints

The monoamine releaser amphetamine has proven effective to decrease human self-administration of cocaine (choice, Refs.^99,100^; clinical trials, Refs.^50,101–103^); however, like many drugs that increase DA, amphetamine has substantial abuse liability. For this reason, we used a low-potency monoamine releaser, phenmetrazine, which is available as a prodrug, phendimetrazine^104^. Phendimetrazine is a very weak monoamine releaser before being metabolized by the liver into phenmetrazine^105–108^. We chose to focus on phenmetrazine in this study since it is the active metabolite of phendimetrazine. Consistent with the results seen in this study, prior studies have also shown that continuous administration of phenmetrazine results in decreased cocaine breakpoints in rodents^60,62^. Decreased choice for cocaine in non-human primates has also been documented (phenmetrazine, Ref.^109^; phendimetrazine,^110,111^). Studies in humans have shown that phendimetrazine is a promising candidate medication for cocaine use disorder. Several report a low abuse potential for phendimetrazine in cocaine-dependent individuals with regard to subjective drug effects such as “drug liking” or “high”^59,112^, in contrast to numerous reports of d-amphetamine increasing abuse-related subjective drug effects^113–116^. It would be interesting to compare the subjective effects of the phenmetrazine prodrug, phendimetrazine, and d-amphetamine’s prodrug, lisdexamfetamine, especially in individuals with cocaine use disorder, but reports of such studies are not available at the present time.

Abuse liability is a significant obstacle to developing drug treatments for substance use disorders and should be weighed carefully against the therapeutic benefit of drugs. Therefore, from a clinical therapeutic perspective, it is advantageous to use prodrugs due to their slow onset of action in the central nervous system, which reduces their abuse liability^117^. In the case of amphetamine and phenmetrazine, more studies are needed to directly evaluate their respective prodrugs, lisdexamfetamine, and phendimetrazine. It is also important to consider DEA schedule status. Phendimetrazine is a schedule III controlled substance, compared to lisdexamphetamine, which is a schedule II substance. This difference in schedule status allows phendimetrazine to be prescribed by physicians under less stringent restrictions, and also increases accessibility for patients since prescription refills are allowed. Although some reports suggest that phendimetrazine may not be highly efficacious on its own in attenuating subjective or reinforcing effects of cocaine^59^, it is possible that phendimetrazine may be successful as part of a dual-target therapy.

### A single injection of nBNI is sufficient to reduce cocaine breakpoints

KORs on DA neurons contribute to the modulation of affective states (for review, see Refs.^118,119^). Particularly, KOR-activation mediated reductions in DA release are thought to contribute to negative affective states^120–122^. Stress induces an increase in dynorphin levels^123,124^, and chronic stress-induced anxiety has been shown to increase cocaine self-administration^125–127^. Stress-induced anxiety-like behaviors are reduced by genetically deleting KORs from DA-containing neurons in mice^118^. Similar to stress, chronic cocaine exposure leads to an upregulation of the KOR system (Refs.^81,83,128,129^, and increases drug-seeking behaviors^77,130^. Additionally, stress-mediated elevations in dynorphin have been shown to reinstate cocaine self-administration in various animal models^75,77,131,132^—an effect which is blocked by KOR inhibition (nBNI, Ref.^132^; JDtic, Ref.^75^). While there have been some studies that have indicated therapeutic benefits of KOR agonists, this effect appears largely dependent on the timing of administration, in addition to context, duration of administration^133–136^, sex^137–139^, and stress history^140,141^, for review see Refs.^142–145^. On the other hand, KOR agonists have also been shown to potentiate reinstatement and self-administration of several drugs (ethanol: Ref.^71,146,147^; cocaine, Refs.^131,133^; nicotine, Ref.^148^). A limitation of this study is the inclusion of only male rats; since sex is known to alter KOR function, our goal was to first delineate the potential role of this dual action of the dopamine and KOR system in male rats.

Notably, a limited number of studies have examined the effects of KOR antagonists on cocaine use and subjective craving in humans^149,150^. Recently, Reed and colleagues (2018) conducted a study assessing the safety and tolerability of the short-acting KOR antagonist LY2456302 (also referred to as JNJ-67953964, CERC-501, OpraKappa, and Aticaprant). LY2456302 was found to be safe and tolerable in early-abstinent, cocaine-dependent individuals; albeit, there was no change in subjective cocaine craving, a secondary outcome of the study^150^. Of note, this study had a small number of subjects, tested only one dose of the KOR antagonist, and only tested effects over 4 consecutive days. Furthermore, this study was not designed to examine LY2456302’s effectiveness to treat cocaine use disorder. More studies are necessary to examine if KOR antagonists are effective at decreasing cocaine self-administration in humans (for review, see Ref.^151^).

### KOR: DAT interactions may underlie added benefits of combination therapy

This study used a combination therapy approach to provide an enhanced profile of anti-cocaine effects. In theory, these dual drugs may individually address two different aspects of the “reward deficit and stress surfeit” syndrome seen in addiction^34,152^. Co-localization of KORs and DATs on presynaptic DA terminals in the NAc allows for functional interactions as well as direct protein–protein binding and complex formation^67,153^. When KORs are activated acutely, the number of KOR-DAT complexes increases, as does the rate of DA uptake through the DAT^69,153^. With repeated KOR agonist treatment, however, sustained decreases in DA uptake have been reported^69^ (but see Ref.^70^). In light of the interaction between KORs and DATs, and the cocaine-induced dysfunction of both the DA and DYN/KOR systems, we chose to determine the pharmacotherapeutic potential of combining the monoamine releaser phenmetrazine and KOR antagonist nBNI.

Combination therapy can provide many benefits over monotherapies, including (1) the ability to use lower doses of drugs, decreasing unwanted side effects compared to single high-dose monotherapy, and (2) the potential for greater overall benefit than either drug alone^154,155^. After two treatment phases, phenmetrazine continued to decrease cocaine breakpoints while nBNI did not. In the group treated with the combination of phenmetrazine and nBNI, cocaine breakpoints continued to decrease to a larger extent than either treatment alone. Additionally, long-term cocaine-induced deficits in DA and KOR systems were returned to control levels by the combination of phenmetrazine and nBNI.

## Conclusions

This is the first study to dual-target the KOR and DAT by administering a monoamine releaser and a KOR antagonist to reduce cocaine self-administration and DA system dysregulations. As clinical treatments become more holistic, it will be important to study combinations of drugs that would be predicted to treat multiple aspects of cocaine addiction, such as drug craving and withdrawal anhedonia, as well as comorbid anxiety or depression. The present results indicate that combining DAT and KOR inhibitors provides a promising avenue for potential medication development. Further investigation is required to understand the physical and functional interactions between the KOR and DAT and how modulation of these cellular targets, together, ultimately influences drug taking behaviors.

## Data Availability

The data generated or analyzed during this study are available from the corresponding author upon reasonable request.
